# Supplementary material for: Epidemiology of soil-transmitted helminths following sustained implementation of routine preventive chemotherapy: Demographics and baseline results of a cluster randomised trial in southern Malawi
Source: PLoS Negl Trop Dis. 2021 May 12;15(5):e0009292. doi: 10.1371/journal.pntd.0009292 (PMC8224978; doi:10.1371/journal.pntd.0009292)
Supplement: S1 Table — (DOCX) [file pntd.0009292.s002.docx]

**Table S1.** Individual and household-level characteristics of parasitological survey participants in total, disaggregated by socio-economic status, and disaggregated by sex; in Namwera, Mangochi district, Malawi in 2018.

|  | **Parasitological survey participants; % (n)** | | | | |
| --- | --- | --- | --- | --- | --- |
|  | **Total:** | **Q1:**  **(Poorest)** | **Q5:**  **(Least poor)** | **Male** | **Female** |
| **Demographic profile:** | | | | | |
| **Age group (years):** | | | | | |
| <1 year | 21.8 (1329) | 23.7 (291) | 19.9 (237) | 28.3 (640) | 18 (689) |
| 1-4 years | 25.3 (1541) | 24.5 (300) | 27 (322) | 33.3 (753) | 20.5 (788) |
| 5-14 years | 52.9 (3228) | 51.8 (635) | 53.2 (635) | 38.3 (867) | 61.5 (2361) |
| Age unknown | 0.07 (4) | 0 (0) | 0 (0) | 0.13 (3) | 0.03 (1) |
| **Individual stayed in household the majority of days in 6 months prior to census survey** | 98.4 (6007) | 99.1 (1215) | 98.2 (1173) | 97.7 (2211) | 98.9 (3796) |
| **Individual slept at household night prior to census survey** | 98.2 (5989) | 98.8 (1211) | 97.2 (1161) | 96.4 (2181) | 99.2 (5989) |
| **Age specific enrolment rate:** | | | | | |
| Age 5 years (OA 1 year before Std 1) | 64.2 (154) | 55 (33) | 88.6 (39) | 70.1 (82) | 58.5 (72) |
| Age 8 years (OA Std 2/3) | 96.8 (152) | 89.7 (26) | 97.3 (36) | 95.4 (83) | 98.6 (69) |
| Age 11 years (OA Std 5/6) | 94 (126) | 88 (22) | 100 (26) | 97.1 (68) | 90.6 (58) |
| Age 14 years (OA Std 8) | 91.3 (94) | 84.2 (16) | 92.3 (24) | 94.2 (49) | 88.2 (45) |
| **Highest level of education (age≥15 years)** | | | | | |
| No formal education | 37.3 (1197) | 49.7 (315) | 25.2 (159) | 23.3 (201) | 42.4 (996) |
| Primary incomplete | 50.4 (1620) | 43.7 (277) | 51.1 (322) | 58.4 (504) | 47.5 (1116) |
| Primary complete or higher | 10.3 (332) | 4.9 (31) | 20.8 (131) | 13.7 (118) | 9.1 (214) |
| Highest education level unknown | 2 (64) | 1.7 (11) | 2.9 (18) | 4.6 (40) | 1 (24) |
| **Dewormed within past 12 months** | 49.3 (3011) | 46.3 (567) | 52.4 (625) | 50.7 (1147) | 48.6 (1864) |
| **Household demographic profile:** | | | | | |
| **Household stayed less than 5 years** | 43.0 (1914) | 35.0 (316) | 43.9 (372) | - | - |
| **Primary language spoken is Chiyao** | 95.9 (4264) | 99.6 (898) | 87.5 (742) | - | - |
| **Primary religion practiced is Islam** | 94.9 (4219) | 99.1 (894) | 86.9 (737) | - | - |
| **Household materials, assets & utilities:** | | | | | |
| **Dwelling has floor of natural material** | 81.0 (3604) | 99.8 (900) | 47.5 (403) | - | - |
| **Household has any livestock** | 39.8 (1768) | 24.8 (224) | 57.4 (487) | - | - |
| **Household has electricity** | 3.5 (157) | 0 (0) | 15.3 (130) | - | - |
| **Household water and sanitation:** | | | | | |
| **Household toilet facility:** | | | | | |
| Basic | 72.3 (3213) | 63.9 (576) | 82.8 (702) | - | - |
| Limited | 21.5 (955) | 26.6 (240) | 14.3 (121) | - | - |
| Unimproved | 4.5 (199) | 5.7 (51) | 2.7 (23) | - | - |
| Open defecation | 1.8 (80) | 3.9 (35) | 0.2 (2) | - | - |
| **Household water source:** | | | | | |
| Basic | 72.4 (3221) | 67.7 (611) | 73.4 (622) | - | - |
| Limited | 24.2 (1077) | 27.6 (249) | 24.2 (205) | - | - |
| Unimproved | 1.3 (58) | 1.8 (16) | 0.8 (7) | - | - |
| Surface water | 0.2 (7) | 0.1 (1) | 0 (0) | - | - |
| Unknown | 1.9 (84) | 2.8 (25) | 1.7 (14) | - | - |

Abbreviations: OA Std=Official age for standard (grade level).
