# Supplementary material for: Epidemiology of soil-transmitted helminths following sustained implementation of routine preventive chemotherapy: Demographics and baseline results of a cluster randomised trial in southern Malawi
Source: PLoS Negl Trop Dis. 2021 May 12;15(5):e0009292. doi: 10.1371/journal.pntd.0009292 (PMC8224978; doi:10.1371/journal.pntd.0009292)
Supplement: Attachment — Submitted filename: Comments to the Author.docx [file pntd.0009292.s004.docx]

Comments to authors:

This study, McManus and colleagues reporting the baseline results of the epidemiology of STH following sustained implementation of routine PCT. The authors included multiple variables related to prevalence, intensity as well as individual, household, water, sanitation and hygiene (WASH) and environmental factors. This is important study, to determine the endemicity level of STH and SCH in the study area as well as their risk factors. The authors have provided the details of the methods and results. They have discussed the results in a balanced way. However, I have made the following general comments to help improve the presentation of the work.

General comments:

1. Abbreviations should only be done once at the first use, please remove instances where you have spelt in full abbreviations more than once. For example abbreviated SAC more than once, lines 81 and 91.
2. Since this study was conducted in an area in Malawi, was it approved by the country’s relevant ethics committee? The ethics numbers provided under subsection “Ethical considerations” seemed to be from research bodies outside the country where the research was conducted.
3. The authors should clarify under the “Methods” section whether they conducted a survey or a census. If both were used, please clarify which segment of the study used which method.
4. Please state how data was collected, paper-based or technology-based, and how that method ensured data quality.
5. Lines 268-269, please clearly state which statistical methods were used to calculate/model prevalence and intensity CI values.
6. Lines 272 and 274, the authors used mixed effects logistic or negative binomial regression to calculate risk factors, which variables were fixed and random?
7. Line 282, regarding community profile, can be summarized in a graphical format to show how the final sampled households and individuals were reached. This should also apply to line 313 on parasitological survey enrolment.
8. Lines 347-365 as well as 376-386, the authors should revise this paragraph(s) by presenting the actual results of the risk factor analysis and not explaining what the results imply.
9. Table 2&3, what does “-” mean? I expect values in those table cells. Also what does “1” indicate, if reference category, then please state so.
