# Supplementary material for: Epidemiology of soil-transmitted helminths following sustained implementation of routine preventive chemotherapy: Demographics and baseline results of a cluster randomised trial in southern Malawi
Source: PLoS Negl Trop Dis. 2021 May 12;15(5):e0009292. doi: 10.1371/journal.pntd.0009292 (PMC8224978; doi:10.1371/journal.pntd.0009292)
Supplement: Attachment — Submitted filename: dw3_mlw_bl_responseletter_20210108.docx [file pntd.0009292.s005.docx]

Friday 8^th^ January 2021

Dear Prof. María-Gloria Basáñez and Aysegul Taylan Ozkan,

We thank you and the reviewers for their comments on this manuscript. Please find our responses to these comments and description of the changes made to the manuscript below in red:

**Attachment ‘Comments to authors’:**

*1. Abbreviations should only be done once at the first use, please remove instances where you have spelt in full abbreviations more than once. For example, abbreviated SAC more than once, lines 81 and 91.*

This has been revised (Introduction, paragraph 2).

*2. Since this study was conducted in an area in Malawi, was it approved by the country’s relevant ethics committee? The ethics numbers provided under subsection “Ethical considerations” seemed to be from research bodies outside the country where the research was conducted.*

This study was approved in Malawi by the College of Medicine Research Ethics Committee (COMREC). The manuscript has been revised (Ethical considerations, paragraph 1) to clarify that COMREC operates from the University of Malawi.

*3. The authors should clarify under the “Methods” section whether they conducted a survey or a census. If both were used, please clarify which segment of the study used which method.*

This manuscript reports a study that consists of a community census followed by a (cross-sectional) parasitological survey. To clarify this, the manuscript has been revised throughout to consistently refer to each activity in these terms.

*4. Please state how data was collected, paper-based or technology-based, and how that method ensured data quality.*

Electronic data collection was used throughout this study. The manuscript has been revised as follows: “…[Census/Survey] questionnaire responses were recorded electronically using the SurveyCTO platform (Dobility, Inc) …” in sub-sections ‘Community census design’ and ‘Parasitological survey design’.

*5. Lines 268-269, please clearly state which statistical methods were used to calculate/model prevalence and intensity CI values.*

As described in the methods section: “…prevalence and intensity descriptive analyses used robust standard errors to account for clustering…” (i.e. when determining CI values). We have added an addition sentence to clarify that “…age- and cluster-population weighted estimates were calculated using the proportion of the censused population living in the cluster…”

*6. Lines 272 and 274, the authors used mixed effects logistic or negative binomial regression to calculate risk factors, which variables were fixed and random?*

As described in the methods section, “…associations between presence of infection and individual, household- and environmental-risk factors were estimated using mixed effects logistic regression, accounting for clustering at the household, village and cluster level…” – this means that random intercepts were included at these levels. We have added additional text to clarify this point.

*7. Line 282, regarding community profile, can be summarized in a graphical format to show how the final sampled households and individuals were reached. This should also apply to line 313 on parasitological survey enrolment.*

The profile of the community census is summarised graphically in the upper half of figure 2 (study participant flow chart) and profile of the parasitological survey in the lower half of figure 2. To clarify the text the first reference to, and placement of, figure 2 has been moved up (Results, paragraph 1) but we would prefer to retain the short text that describes this figure.

*8. Lines 347-365 as well as 376-386, the authors should revise this paragraph(s) by presenting the actual results of the risk factor analysis and not explaining what the results imply.*

Whilst we agree with the reviewer that results sections should only state findings, with interpretations left to the discussion, we only present the direction of associations within the results text – values are not provided as this would simply repeat what is provided in the results tables.

*9. Table 2&3, what does “-” mean? I expect values in those table cells. Also what does “1” indicate, if reference category, then please state so.*

The “-” character is to indicate that the cell is intentionally blank rather than missing. These cells are intentionally blank as only one p-value is reported for each categorical variable (joint significance test) and/or that the variable was not included in the final multivariable model. The “1” character does indicate the reference category. This has now been clarified (footnote 2, tables 2 and 3).

**Attachment ‘PNTD-D-20-01836_reviewer’:**

Formatting revisions recorded on this *.pdf version of the manuscript have been updated in the *.docx format manuscript attached to this submission. Please note that although figure 1 and figure 2 have not been revised, as they have switched position in the text they have been re-attached to this submission with the correct titles and file names.

**Email body ‘Reviewers response to questions’:**

Results: Reviewer #1: *I wonder why there is no P-value mentioned in the Univariable analysis for Household flooring (both in prevalence and intensity column).*

This was erroneous and has now been corrected. Some additional minor corrections have also been made to the values in table 3 (intensity).

Yours sincerely,

Stefan Witek-McManus
